# Whole genome sequencing and analysis of multiple isolates of *Ceratocystis destructans*, the causal agent of Ceratocystis canker of almond in California

**DOI:** 10.1038/s41598-023-41746-6

**Published:** 2023-09-08

**Authors:** Tawanda E. Maguvu, Renaud Travadon, Dario Cantu, Florent P. Trouillas

**Affiliations:** 1 Department of Plant Pathology, University of California, Davis, CA 95616 USA; 2 Kearney Agricultural Research and Extension Center, Parlier, CA 93648 USA; 3 Department of Viticulture and Enology, University of California, Davis, CA 95616 USA

**Keywords:** Genomic analysis, Next-generation sequencing, Fungal genomics, Fungal pathogenesis

## Abstract

Ceratocystis canker caused by *Ceratocystis destructans* is a severe disease of almond, reducing the longevity and productivity of infected trees. Once the disease has established in an individual tree, there is no cure, and management efforts are often limited to removing the infected area of cankers. In this study, we present the genome assemblies of five *C. destructans* isolates isolated from symptomatic almond trees. The genomes were assembled into a genome size of 27.2 ± 0.9 Mbp with an average of 6924 ± 135 protein-coding genes and an average GC content of 48.8 ± 0.02%. We concentrated our efforts on identifying putative virulence factors of canker pathogens. Analysis of the secreted carbohydrate-active enzymes showed that the genomes harbored 83.4 ± 1.8 secreted CAZymes. The secreted CAZymes covered all the known categories of CAZymes. AntiSMASH revealed that the genomes had at least 7 biosynthetic gene clusters, with one of the non-ribosomal peptide synthases encoding dimethylcoprogen, a conserved virulence determinant of plant pathogenic ascomycetes. From the predicted proteome, we also annotated cytochrome P450 monooxygenases, and transporters, these are well-established virulence determinants of canker pathogens. Moreover, we managed to identify 57.4 ± 2.1 putative effector proteins. Gene Ontology (GO) annotation was applied to compare gene content with two closely related species *C. fimbriata*, and *C. albifundus*. This study provides the first genome assemblies for *C. destructans*, expanding genomic resources for an important almond canker pathogen. The acquired knowledge provides a foundation for further advanced studies, such as molecular interactions with the host, which is critical for breeding for resistance.

## Introduction

**Figure 1:**
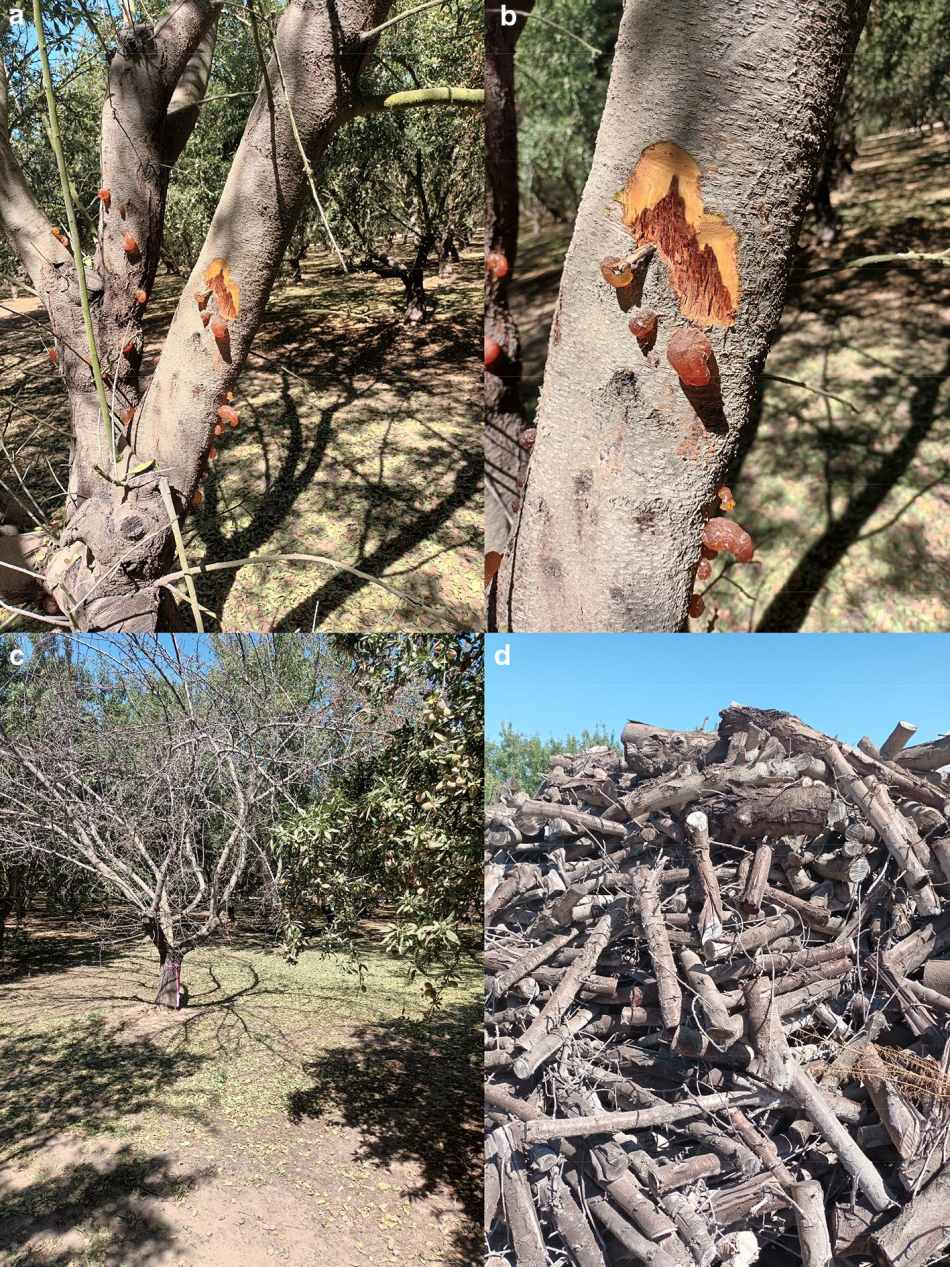
Symptoms and impacts of Ceratocystis canker on almonds. Ceratocystis canker developing near large pruning wounds (**a**). A closer look at an active Ceratocystis canker extending from a pruning wound site, with amber-colored gum balls developing at the canker margin (**b**). Dieback of an entire almond tree resulting from Ceratocystis canker which girdled the trunk (**c**). A pile of almond logs removed from Ceratocystis canker infested trees from an orchard (**d**).

Ceratocystis canker of almond, also known as the mallet wound canker, is caused by the ascomycete fungal pathogen *Ceratocystis destructans*. The disease mainly develops on areas of the trunk or branches that have been damaged by tractors, hedgers, harvesting equipment, and at pruning wounds. Ceratocystis canker shorten the life span and productivity of affected trees, overall reducing the orchard’s profitability. Symptoms of Ceratocystis canker include water-soaked lesions with amber-colored gum balls developing at the canker margin (Fig. 1). With time, cankers may girdle and kill infected branches or trunks. *C. destructans* is spread by several species of sap-feeding insects, including fruit flies and beetles, which either ingest fungal spores and later excrete them or transport them on their bodies to new locations^1,2^. Wounds on almond trees are susceptible to *Ceratocystis* for about 8 to 14 days, thereafter the chances of infection are greatly reduced^3^. Once spores are spread to fresh almond wounds and the new canker establishes, there is no cure for the infected tree. The most effective way to manage Ceratocystis cankers is to avoid injuries to trunks and scaffold branches. Tree surgery can be used eventually in an attempt to remove the infected area of cankers. However, this technique is tedious and rarely successful, in which case the fungus continues to grow. Recently, Holland et al.^4^ reported a widespread occurrence of Ceratocystis canker, which appears ubiquitous in almond-producing counties of California. The same authors concluded that *C. destructans* is likely endemic to California, occurring naturally on the native vegetation surrounding orchards^5^.

To colonize woody plants, trunk pathogens rely on carbohydrate active enzyme (CAZymes), as they are responsible for the breakdown of plant cell wall components (cellulose, hemicellulose, xylan, xyloglucan, mannan and pectin)^6,7^. Owing to their involvement in plant cell wall degradation during the colonization of plants by phytopathogens, CAZymes are an important virulence factor of phytopathogens^8,9^. Besides CAZymes, several secondary metabolites and their toxins, membrane transporters, and cytochrome P450 monooxygenases have been shown to be critical virulence factors in most canker pathosystems^10–13^. Cytochrome P450s facilitate fungal adaptations to various ecological niches by buffering harmful chemical and biological cues^13,14^. They also play a critical role in the post-synthesis modification of diverse metabolites^14^. Transporters such as the major facilitator superfamily (MFS) are single-polypeptide secondary carriers capable of transporting small solutes in response to chemiosmotic ion gradients. These can play a crucial role in antifungal resistance and nutrient transport^15,16^, which is crucial for pathogenesis in response to reactive oxygen species. Such a wealth of knowledge about virulence factors of phytopathogenic fungi can be exploited to infer hypothetical proteins involved in the pathogenesis and or virulence of new species. And this can be built-upon for further studies, such as pathogen-host interactions, contributing to advanced understanding of emerging/new phytopathogens.

Advances in whole genome sequencing along with many bioinformatics computational tools offer an unprecedented capability to generate and mine genomic sequences of plant pathogens^17^. The fungal secretome is the set of proteins that contain a signal peptide and are processed via the endoplasmic reticulum and golgi apparatus, among which effectors help circumvent host defenses by either masking the presence of the pathogen or killing the host cell directly^18,19^. Effectors and the other established virulence factors of canker pathogens can be mined from genome sequences. Several bioinformatics tools which can be integrated to predict fungal virulence factors from genomic sequences are readily available. These include but are not limited to SECRETOOL^20^, CAZy database^21^, antiSMASH^22^, Fungal cytochrome P450 database (FCPD)^23^, SignalP^24^, and effectorP^25^. The wealth of information obtained from these and other tools provides a baseline for experimental work, such as understanding how pathogens colonize the host, leading to a better solution for the control and management of plant pathogens.

In the present study, we present high-quality genome assemblies of five *C. destructans* isolates (KARE490, KARE979, KARE1428, KARE1622, and KARE1624) obtained from canker-infested almond (*P*. *dulcis*) trees in California. The main differences among the isolates were the geographical location from which they were isolated, albeit all of them were from the San Joaquin Valley, California. For precise location details, refer to^5^. Currently, there are no publicly available *C. destructans* genomes, and this report presents the first comprehensive genomic resource of an important almond canker pathogen. The study also presents descriptions of the potential roles of some of the predicted virulence factors. To benchmark the analysis of our genome sequences, we included the genomes of *C. fimbriata* and *C. albifundus*, two well-established *Ceratocystis* species with available genomic information. *C. fimbriata*, the type species of the genus *Ceratocystis*, causes black rot of sweet potato as well as wilt or cankers in rubber trees, coffee, and *Eucalyptus* spp.^26^. *C. albifundus* is an aggressive tree pathogen native to Africa, mainly known as the causal agent of Ceratocystis wilt in *Acacia mearnsii*^27^. The genome of *C. albifundus* has been sequenced several times, and it is well annotated and less fragmented, making it good for benchmarking our analysis.

## Results and discussion

### Genome sequencing, assembly, and protein prediction

Genomes of the *Ceratocystis destructans* isolates were sequenced with Illumina HiSeq2500 and assembled to a genome size of 27.2 ± 0.9 Mbp organized into 663.2 ± 34.1 scaffolds having an N50 of 179.96 ± 18.9 Kbp (Table 1). Table 1 provides detailed assembly and quality metrics for each of the five sequenced isolates (KARE979, KARE1622, KARE1624, KARE1428, and KARE490). The relatively small genome size of *C. destructans* isolates is in congruence with the genome sizes of other published *Ceratocystis* genomes^28–32^, Table 1. Assessment of genome integrity by using BUSCO analyses^33^ showed that most of the conserved core fungi genes were predicted (> 97%; Table 1), which indicates that our genomes were almost complete. The total predicted protein-coding genes for the *C. destructans* isolates, and those for *C. albifundus* and *C. fimbriata* are shown in Table 2. The number of the total predicted protein-coding genes in *C. destructans* did not greatly differ from those of *C. albifundus* and *C. fimbriata*, another indication of good quality for our newly sequenced genomes (Table 2). The relatively smaller size of the *C. destructans* genomes compared to most canker pathogens like *Eutypa lata, Neofusicoccum spp*., and *Diplodia spp*. correlated with their small number of predicted protein-coding genes, which is consistent with previous reports on the genomes of other *Ceratocystis* species^28–32^. Though we combined two algorithms, Augustus, and GeneMark-ES for gene predictions, integrating *C. destructans* transcripts in the analyses will likely improve the protein-coding gene prediction. Integration of RNA-seq data has been shown to significantly improve protein-coding gene prediction in eukaryotes^12,34,35^. Thus, predictions from this study could be further refined in the future by integrating RNA-seq data, using *C. destructans* transcripts obtained under a range of experimental conditions. Moreover, *C. destructans* transcripts in planta would be a very useful resource and might lead to the identification of candidate pathogenicity genes that are only expressed when the pathogen is growing within host tissues, as was recently observed for an *Ophiostoma novo-ulmi* ortholog of gene *AOX1* encoding an alcohol oxidase^36^. Analysis of *C. destructans* transcripts at different time points during the colonization of almond tissue could further enhance our knowledge of *C. destructans* genes expressed when the pathogen is growing within host tissue.

**Table 1:** Assembly and quality metrics of the *Ceratocystis destructans* isolates and the related *Ceratocystis* genomes.

Species	Assembly size (Mb)	Scaffolds	Scaffold N50 (Kb)	Scaffold L50 count	BUSCO estimated completeness	GC %
*C. destructans* (KARE1622)	27.6	688	148.6	59	99.1	48.83
*C. destructans* (KARE490)	27.6	654	187.6	48	98.2	48.85
*C. destructans* (KARE979)	27.6	702	187.2	42	97.9	48.83
*C. destructans* (KARE1624)	27.7	614	178.2	45	99.1	48.8
*C. destructans* (KARE1428)	25.6	658	198.2	48	98.9	48.83
*C. fimbriata*	31.1	390	184.3	49	98.7	48.1
*C. albifundus*	29.2	38	2308.2	5	97.9	48.98

**Table 2:** Protein-coding genes predicted as secreted CAZymes, or part of a gene cluster encoding secondary metabolites.

Species	Total predicted genes	Secreted CAZymes	Secondary metabolites gene clusters					
			Terpene	NRPS	NRPS-like	T1PKS	T3PKS	RiPP
*C. destructans* (KARE1622)	7036	83	**1** (pimara-8(14),15-diene)	**2** (dimethylcoprogen; choline)	**1** (basidioferrin)	**1** (8-methyldiaporthin)	**1** (unknown)	**2** (megacin; asperipin 2a)
*C. destructans* (KARE490)	6722	86	**1** (pimara-8(14),15-diene)	**2** (dimethylcoprogen; choline)	**1** (basidioferrin)	**1** (8-methyldiaporthin)	**1** (unknown)	**1** (megacin)
*C. destructans* (KARE979)	6850	81	**1** (pimara-8(14),15-diene)	**2** (dimethylcoprogen; choline)	**1** (basidioferrin)	**2** (8-methyldiaporthin; depudecin)	**1** (unknown)	**2** (megacin; asperipin 2a)
*C. destructans* (KARE1624)	7021	83	**1** (pimara-8(14),15-diene)	**2** (dimethylcoprogen; choline)	**1** (basidioferrin)	**2** (8-methyldiaporthin; depudecin)	**0** (absent)	**2** (megacin; asperipin 2a)
*C. destructans* (KARE1428)	6993	84	**1** (pimara-8(14),15-diene)	**2** (dimethylcoprogen; choline)	**0** (absent)	**2** (8-methyldiaporthin; depudecin)	**1** (unknown)	**1** (megacin)
*C. fimbriata*	7785	96	**1** (pimara-8(14),15-diene)	**2** (dimethylcoprogen; choline)	**1** (basidioferrin)	**2** (8-methyldiaporthin; 6-methylsalicyclic acid)	**1** (abscisic acid)	**3** (megacin; asperipin 2a; SCO-2138)
*C. albifundus*	7179	74	**1** (pimara-8(14),15-diene)	**1**(dimethylcoprogen)	**2**(virginiafactin; livipeptin)	**2** (8-methyldiaporthin; citreoviridin)	**1** (terretonin)	**3** (megacin; Type R and F tailocins; SCO-2138)

Compounds predicted to be encoded by the BGCs using the Minimum Information about the Biosynthetic Gene Cluster (MIBiG) database are shown in parenthesis

Bold numbers indicates total predicted BGCs per category.

### Gene content comparison with a focus on pathogenesis-related features

**Figure 2:**
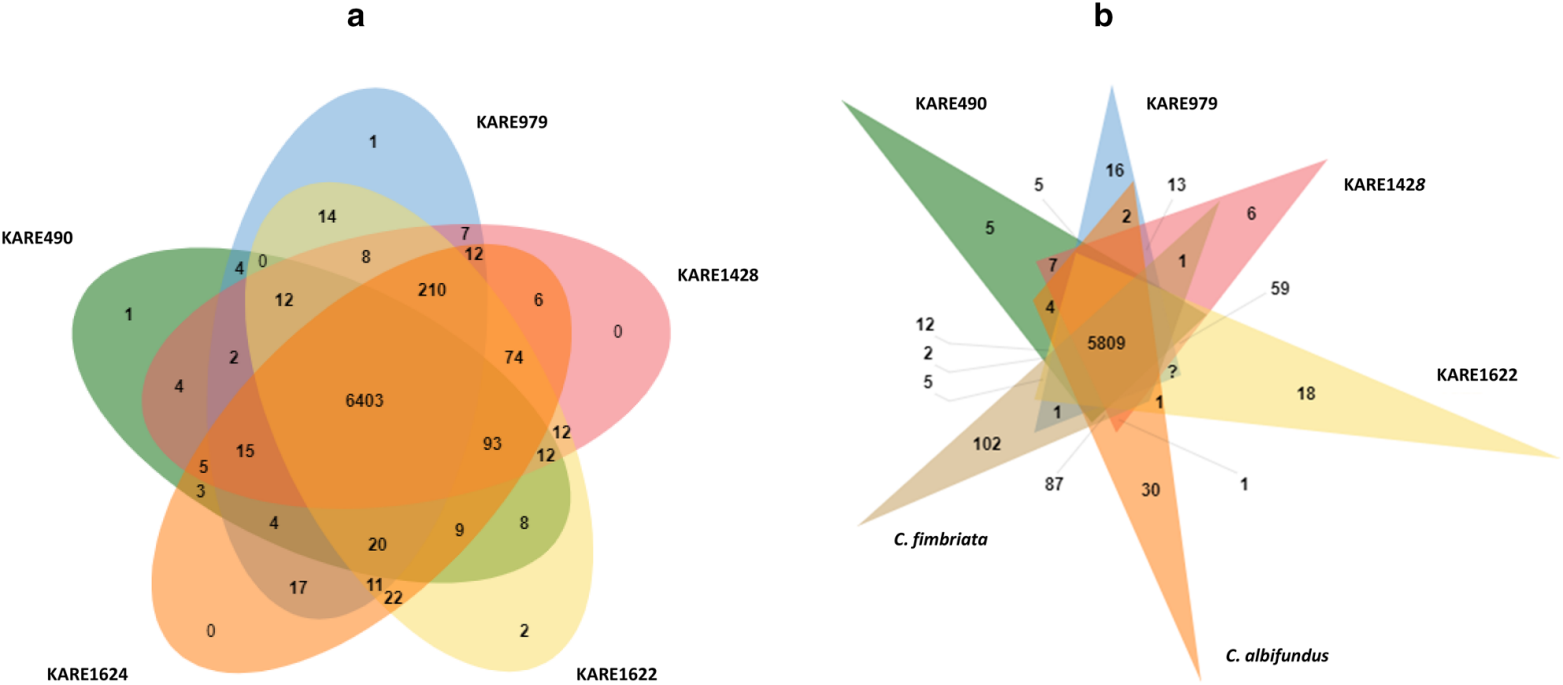
Venn diagrams showing the distribution of shared orthologous clusters among the *Ceratocystis* genomes. Shared orthologous clusters among *C. destructans* isolates (**a**). Shared orthologous clusters among *C. fimbriata*, *C. albifundus* and the *C. destructans* isolates (**b**).

The *C. destructans* isolates shared 6,403 orthologous genes (Fig. 2A). 64. 2% (4, 110) of the shared orthologous genes had a GO annotation, of which only 33 gene clusters had a “pathogenesis” GO annotation. These included 1-phosphatidylinositol phosphodiesterase (14), major facilitator superfamily multidrug transporter NAG4, sucrose non-fermenting protein kinase 1, dipeptidase sirJ, probable aspartic-type endopeptidase, D-arabinitol dehydrogenase 1, transcription activator AMTR1, acyl-CoA ligase sidI, calcium channel YVC1, ferric/cupric reductase transmembrane component 1, C2H2 finger domain transcription factor (mtfA and dvrA), beta-1,2-xylosyltransferase 1, bZIP transcription factor hapX, bZIP-type transcription factor MBZ1, phospho-2-dehydro-3-deoxyheptonate aldolase AMT16, initiation-specific alpha-1,6-mannosyltransferase, and carboxypeptidase S1 homolog. Pathogenesis GO annotations were supplemented by querying the pathogen-host interaction database (PHI-base). All the proteins with a pathogenesis GO annotation had a hit in the PHI-base except for beta-1,2-xylosytransferase 1 (Supplementary data 1). Supplementary data 1 also provides the phenotypes of the top hits in the PHI-base, and the phenotypes of the other hits.

A very high copy number of 1-phosphatidylinositol phosphodiesterase orthologous group have also been reported in other *Ceratocystis* genome studies. The evolution of this gene family is surmised to be guided by positive selection^31,32^, suggesting that this family is critical for the *Ceratocystis* genus. Phosphatidylinositol-specific phospholipases are responsible for cleaving glycerophospholipids containing the polar head, and they have been associated with functions such as pathogenicity and release of glycosyl-PI (GPI) anchored surface proteins from target membranes^37–39^. GPI-anchored proteins play an important role in the virulence of fungal pathogens by serving as adhesins and variable epitopes to evade the immune system^40^. In *Magnaporthe oryzae*, deletion of the fungal *phospholipase C* gene suppressed calcium flux resulting in defective appressorium formation and pathogenicity^41^. With the evolution of the phosphatidylinositol phosphodiesterase surmised to be guided by positive selection, it will be interesting to conduct *in planta* transcriptomics and functional deletion studies that might shed more information into the role of *C. destructans* 1-phosphatidylinositol phosphodiesterase family. Recently, a few protocols have been developed for successfully genetic manipulation of *Ceratocystis* spp. that opens the door for possibilities of producing knockout mutants in *C. destructans*. These include agrobacterium-based transformation^42,43^ and the CRISPR based method^44^.

When phytopathogenic fungi invade plants, the production of reactive oxygen species at the infection site is the earliest response of pathogen-associated molecular pattern triggered immunity (PTI). This triggers a cascade of effects such as cell wall strengthening, induction of antimicrobial activity, synthesis of pathogenesis-related proteins and phytoalexins, and programmed cell death^45^. However, fungal pathogens have developed a broad arsenal of effector proteins to antagonize plant response to infection. Through D-arabinitol dehydrogenase 1, they can produce D-arabinitol which is capable of quenching reactive oxygen species involved in host plant defense reactions, thus protecting the fungus during the pathogenic interaction^46^. Moreover, transporters such as the major facilitator superfamily (MFS), which are single-polypeptide secondary carriers capable of transporting small solutes in response to chemiosmotic ion gradients, can play a crucial role in antifungal resistance and nutrient transport^15,16^. This is crucial for pathogenesis in response to reactive oxygen species. Null mutations in *Candida albicans* major facilitator superfamily multidrug transporter NAG4 (*CaNAG4*) resulted in increased susceptibility to cycloheximide and attenuated virulence in mice^16^. The calcium channel YVC1 is another important element that plays a critical role in response to stress and contributes to virulence in fungi. *In C. albicans*, the *yvc1*Δ/Δ mutant displayed the decreased ability of stress response, morphogenesis, and attenuated virulence, the Yvc1 was shown to have a role in hyphal polarized growth and re-orientation to host signals^47^. Calcium signaling plays a critical role in fungi’s response to environmental stimuli which includes calcium depletion, alkaline pH, and antifungal compounds. Such environmental stimuli activate plasma membrane-localized high-affinity calcium uptake system resulting in the activation of calcineurin which then triggers the activation of calcium-dependent responses such as cell wall integrity genes^48–50^. The presence of such strong mechanisms to go pound for pound in response to the host antagonizing colonization by phytopathogenic fungus shows how the pathogens are continuously evolving in the arms race.

Orthologues for D-arabinitol dehydrogenase 1, major facilitator superfamily multidrug transporter NAG4, and the calcium channel YVC1 were detected in the genome sequences of *C. destructans.* This provides a brief insight into how *C. destructans* can successfully colonize almond. Also detected was sucrose non-fermenting protein kinase 1, a gene that activates the expression of galactose oxidase, as well as that of several cell-wall degrading enzymes^51^. Plant cell walls provide constitutive barriers that plant pathogens must overcome before accessing cellular components^52,53^, thus activation of fungal cell wall degrading enzymes is crucial for pathogenesis. In *Fusarium virgulifome* the causal organism of sudden death syndrome (SDS) in soybean, disruption of the sucrose non-fermenting protein kinase 1 gene (*FvSNF1*) resulted in severely impaired ability to cause SDS^51^.

BZIP-type transcription factor MBZ1, a gene that plays a crucial role in cell wall integrity, adherence, and virulence, was also present in the orthologous genes shared by genome sequences of *C. destructans.* In *Metarhizium robertsii,* a deletion mutant of the MBZ1 resulted in defects in cell wall integrity, adhesion to hydrophobic surfaces, and infection of the host^54^. Another gene that plays a key role in cell wall integrity and host colonization which was present in the *C. destructans* genomes is ferric/cupric reductase transmembrane component 1. In *C. albicans*, disruption of CFL1 resulted in hypersensitivity to chemical and cell wall stresses moreover, the mutant displayed a decreased ability of adhesion and invasion of host epithelial cells, clearly indicating its role in pathogenesis^55^. The C2H2 finger domain transcription factor mtfA is also a well-described virulence factor. Its deletion in *Aspegillus fumigatus* resulted in decreased virulence, a decrease in the secondary metabolite gliotoxin, and attenuated protease activity^56^, a clear indication of its role in pathogenesis. Another transcription factor in the genome sequences of *C. destructans*, bZIP transcription factor hapX, has been shown to play a role in the expression of genes involved in reductive iron assimilation and siderophore-mediated iron uptake, a process that was shown to be essential for maximum virulence in *A. fumigatus*^57^.

When *C. albifundus* and *C. fimbriata* were added to the analysis, there was a total of 5,809 shared orthologous genes (Fig. 2B). Of the shared orthologous genes among *C. albifundus*, *C. fimbriata*, and *C. destructans* isolates, 30 had a “pathogenesis” GO annotation, and they were the same as those described for *C. destructans*.* C. albifundus* had 30 singleton/unique proteins (Fig. 2B) of which none of them had a “pathogenesis” GO annotation. In contrast, *C. fimbriata* had 102 singletons/unique proteins (Fig. 2B), and only two had a “pathogenesis” GO annotation, both were 1-phosphatidylinositol phosphodiesterase. 495 proteins were unique to at least 2 of the *C. destructans* isolates. Annotations of the proteins identified proteins 1-phosphatidylinositol phosphodiesterase, and patatin group M-2 as proteins that might play a crucial role in the adaptation of *C. destructans* to almond. Querying the PHI-base produced top hits with reduced virulence phenotypes, indicating their possible role in the infection of almond. These protein sequences are provided, in Supplementary Data 1, and their expression profiles during the colonization of almond tissues may shed more light on their roles. Though the numbers of genes with GO annotation that included “pathogenesis” did not drastically differ among *Ceratocystis* species, it is likely that the unique association of *C. destructans* with almond is the result of a genetic adaptation. Even in species with high genome similarity, host specificity might be related to variations in a single locus or clusters of closely linked loci^58^. They were a few more variations observed when *C. destructans* genomes were compared to *C. albifundus* and *C. fimbriata,* a possible explanation for *C. destructans* adaptation to almonds. Most notably, 15 predicted putative effector proteins from *C. destructans* formed monophyletic clusters indicating they were distinct from those in *C. albifundus* and *C. fimbriata* (Supplementary Fig. 2). All of them had a hit in the PHI-database (Supplementary data 1) and could be linked with the specific adaptation of *C. destructans* to almond. Using BLASTP on the NCBI database, they were annotated as hypothetical proteins (12), no hits (2), and putative alpha-1,2-galactosyltransferase (Supplementary data 1). Moreover, CAZymes glycoside hydrolases (GH32, GH5) and an unknown secondary metabolite gene cluster showed some variations among *C. fimbriata, C. albifundus,* and *C. destructans isolates*. (Fig. 4, Table 2). In addition, the overall nucleotide identity was also distinct among the *Ceratocystis* species used in this study. The *C. destructans* isolates shared a minimum of 99% average nucleotide identity. In contrast, they shared 89–92% average nucleotide identity with *C. fimbriata* and *C. albifundus* (Supplementary Fig. 1). Moreover, slight variations could also be noticed in the total number of proteomes predicted as putative effectors, cytochrome P450s, and transporters (Table 3). Cytochrome P450s have been shown to be pivotal in genotypic and phenotypic evolution, understanding the ecological specialization of fungal species^59,60^.

**Table 3:** Number of protein-coding genes annotated in the highlighted functional categories.

Species	Total predicted P450s	Total predicted secretome	Total Predicted Effectors	Total predicted transporters
*C. destructans* (KARE1622)	46	201	59	254
*C. destructans* (KARE490)	37	189	54	252
*C. destructans* (KARE979)	38	196	58	248
*C. destructans* (KARE1624)	41	201	57	251
*C. destructans* (KARE1428)	42	204	59	251
*C. fimbriata*	121	277	104	247
*C. albifundus*	39	191	62	241

**Figure 3:**
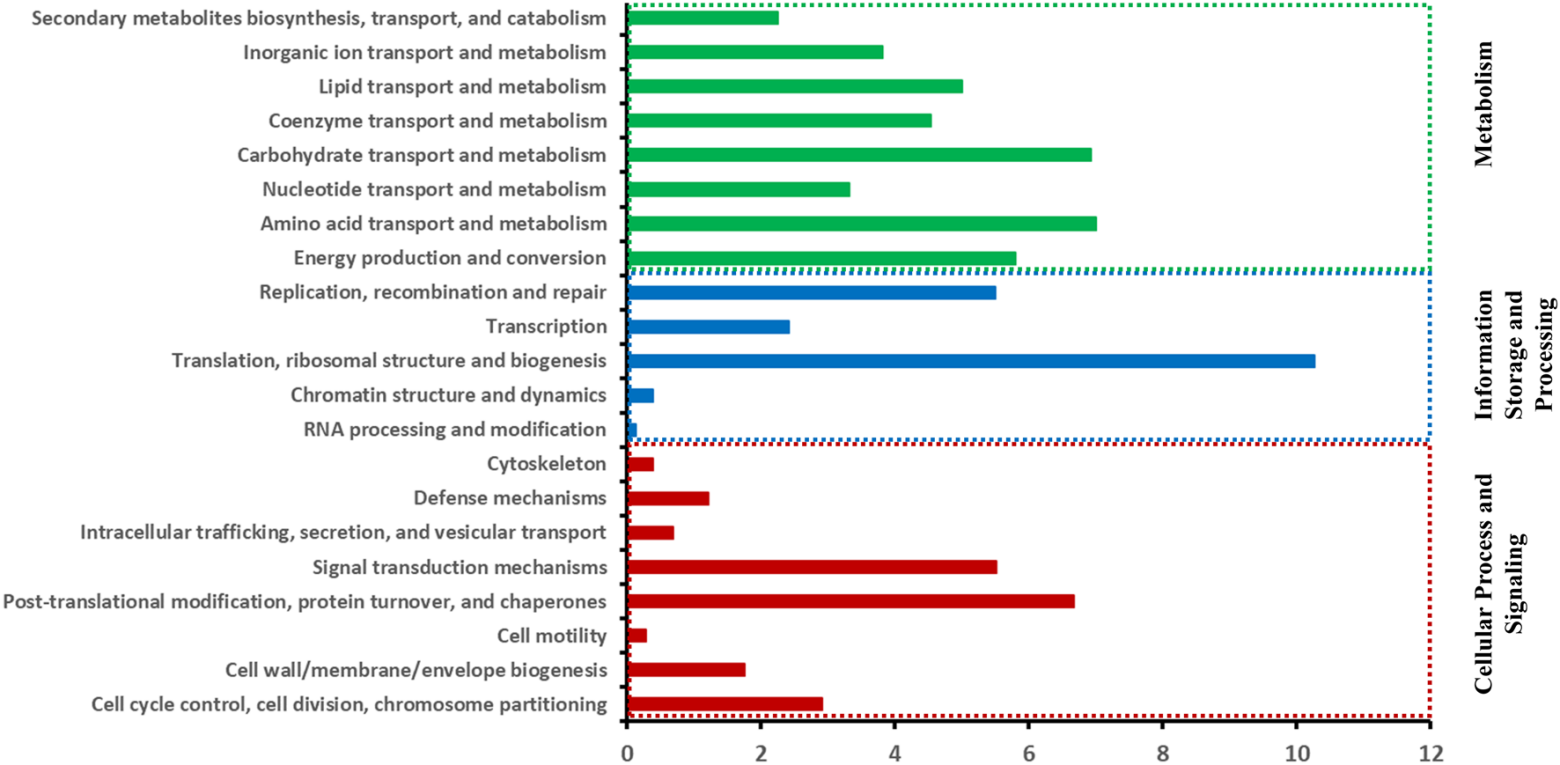
Distribution of the characterized functional categories of the clusters of orthologous genes (COG). The figure shows isolate *C. destructans* (KARE490) and represents the general distribution of the functional categories in the other isolates. Specific information for the individual isolates is provided in Supplementary Data 1. The X-axis represent the % contribution to the total predicted proteome of the genome.

The distribution of the functional categories of the predicted clusters of orthologous genes is shown in Fig. 3 and Supplementary Data 1. In the cellular process and signaling category, the most common assignments were post-translational modification, protein turnover, and chaperones (6.674%), signal transduction mechanisms (5.52%), and cell cycle control, cell division, chromosome partitioning (2.91%) (Fig. 3 and Supplementary Data 1). In the information storage and processing category, largest classes were translation, ribosomal structure, and biogenesis (10.27%), replication recombination and repair (5.49%), and transcription (2.42%) (Fig. 3 and Supplementary Data 1). In the metabolism category, amino acid transport and metabolism (7%), carbohydrate transport and metabolism (6.92%), energy production and conversion (5.8%), and lipid transport and metabolism (5%) were some of the most common categories (Fig. 3 and Supplementary Data 1).

### Carbohydrate active enzymes

**Figure 4:**
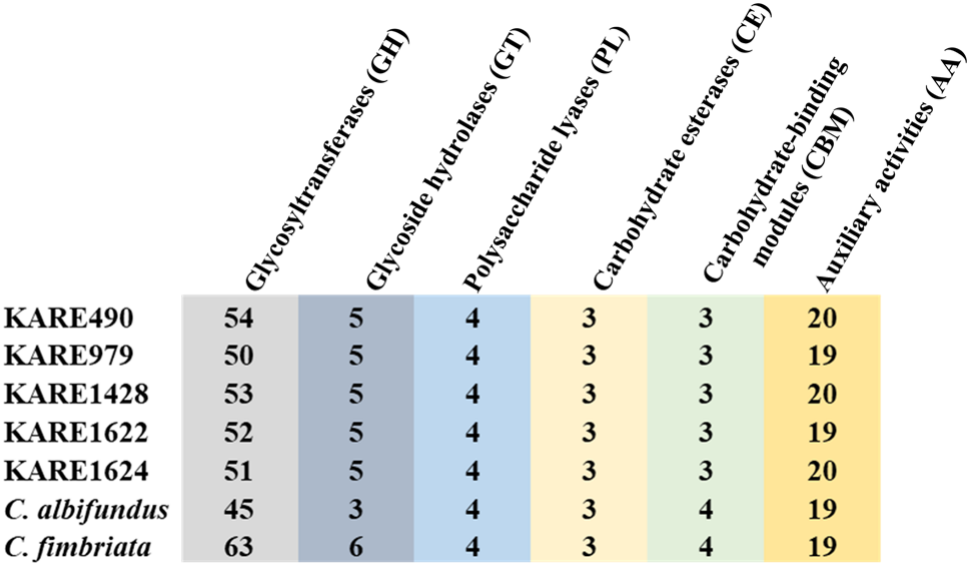
Distribution of secreted CAZymes families among *C. destructans*, *C. fimbriata*, and *C. albifundus.*

The *C. destructans* genomes had 83.4 ± 1.8 secreted CAZymes, in contrast, *C. albifundus* and *C. fimbriata* had 74 and 96, respectively (Table 2). The total number of secreted CAZymes in *C. destructans* was comparable to those of *C. manginecans* and *C. fimbriata* reported by Fourie et al.^32^. In that study, *C. fimbriata* was predicted to have 83 secreted CAZymes while in our current study, a total of 96 secreted CAZymes was predicted. Such slight variation can be attributed to several factors such as slight differences in bioinformatics analysis, as well as advancement in databases. But overall, this indicates that our analysis is comparable to other studies. In the *Ceratocystis* genomes, the glycoside hydrolases (GHs) superfamily contributed to more than 50% of the secreted CAZymes (Supplementary Data 2). The top two GHs of all the analyzed *Ceratocystis* genomes consisted of GH16, and GH18 (Supplementary Fig. 3), which are involved in the degradation of cellulose and hemicellulose^61^. Figure 4 shows the distribution of the secreted CAZyme families and how they correlated among the genomes. Among the most abundant CAZymes, the Auxillary Activities (AA) family was of interest due to *P. dulcis* being a woody plant, thus its cell wall includes lignin; and AAs decompose lignin. The most abundant were AA1, AA7, and AA9 (Supplementary Fig. 3). The AA1s are multicopper oxidases, that catalyze the one-electron oxidation of phenolics, aromatic amines, and other electron-rich substrates via the reduction of oxygen to water^62^. They are involved in a diverse range of functions which includes lignin degradation, and offensive and defensive fungal interaction strategies^63,64^. AA7s consist of gluco-oligosacharide oxidases that generate hydrogen peroxide which is used by peroxidases during the decomposition of lignin^65^. The AA9 family consists of lytic polysaccharide monooxygenases, which degrade cellulose thus rendering it accessible to other CAZymes^66^. The distribution of the other CAZymes is shown in Fig. 4 and Supplementary Data 2. All these works synergistically to optimize the fitness of the phytopathogens.

The plant cell wall is composed of carbohydrates such as pectin, cellulose, and hemicellulose, structural proteins, as well as aromatic compounds^67,68^. The structures that form the plant cell wall are part of a constitutive barrier that plant pathogens must overcome before accessing cellular components^52,53^. Carbohydrate-active enzymes (CAZymes) consist of proteins with the capability to degrade, modify, or create glycosidic bonds^61^. They are divided into four main classes: glycoside hydrolases (GH), polysaccharide lyases (PLs), Carbohydrate esterases (CEs), and glycotransferases^61^. Owing to their involvement in plant cell wall degradation during the colonization of plants by phytopathogens, CAZymes are an important virulence factor of phytopathogens^8,9^. Since fungal effector proteins are generally secreted, CAZymes annotations are always combined with signal peptide prediction to ascertain their probable roles as virulence factors. Not all members of the CAZymes are secreted proteins, as a result, studies aiming to identify effector proteins have always combined CAZymes annotations with the prediction of secreted proteins^12,69^. The *Ceratocystis* genomes used in this study had the secreted CAZymes which covered all the classes (Fig. 4, Supplementary Data 2), an indication they are equipped to bridge the constitutive barrier presented by the host plant.

### Secondary metabolite gene cluster

Fungal secondary metabolites play a key role in mediating ecological interactions, communication, nutrient acquisition, stress protection, and chemical warfare^70^. In phytopathogens like *Stemphylium lycopersici*, the release of some secondary metabolites resulted in necrosis of plant tissue suggesting their probable role as determinants of pathogenicity and virulence^19,71,72^. Genes encoding secondary metabolites are usually clustered together on chromosomes. This observation led to the emergence of genome mining as a technology to identify biosynthetic gene clusters (BGCs) which encode secondary metabolites^73^. In the current study, we took advantage of the advances in both DNA sequencing and secondary metabolites genome mining tools, including antiSMASH^22^ and the Minimum Information about the Biosynthetic Gene Cluster (MIBiG)^74^ to explore the secondary metabolites encoded by the genomes of *C. destructans*. Analysis with antiSMASH revealed the presence of seven secondary metabolites gene clusters in *C. destructans* isolates KARE490, and KARE1428, and eight secondary metabolites gene clusters in isolates KARE1624 and KARE1622 (Table 2). Isolate KARE979 had nine secondary metabolite gene clusters whereas *C. albifundus* and *C. fimbriata* had a total of ten secondary metabolite gene clusters (Table 2). The categories of the biosynthesis gene clusters included type I polyketide synthase (T1PKS), type III polyketide synthase (T3PKS), non-ribosomal peptide synthase (NRPS), non-ribosomal peptide synthase-like (NRPS-like), and ribosomally synthesized and post-translationally modified peptides (RiPPs) (Table 2).

**Figure 5:**
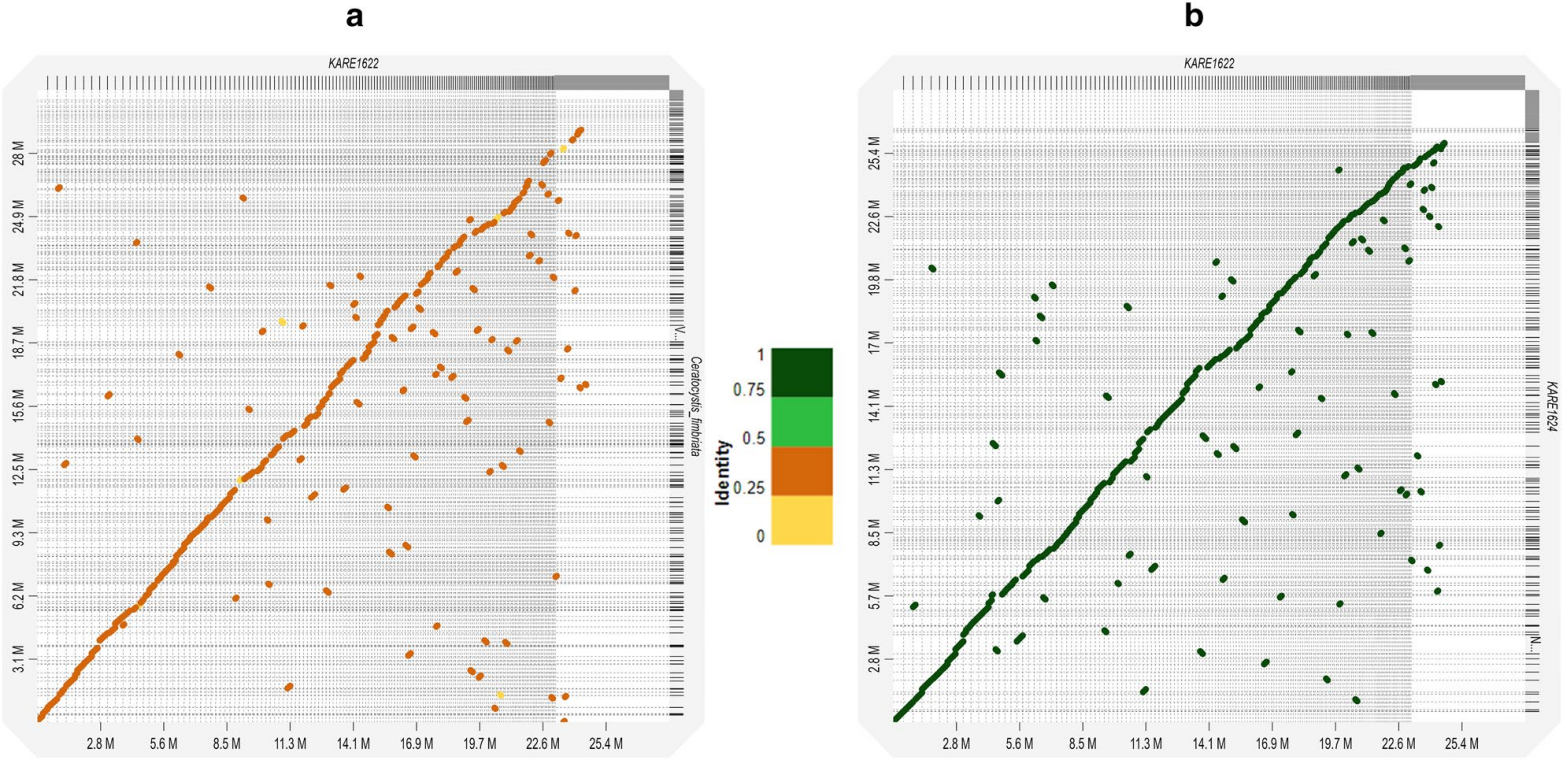
Dot plot graph showing syntenic blocks between *Ceratocystis* genomes. Syntenic blocks between *C. destructans* (KARE1622) and *C. fimbriata* (**a**). Syntenic blocks between C. destructans isolates KARE1622 and KARE1624 (**b**).

Table 2 provides information on the likely compound linked to each BGC based on the MIBiG. Compounds linked to the *C. destructans* isolates BGCs were similar except that the isolates with fewer BGCs lacked one or two compounds in comparison to isolates with more BGCs (Table 2). The slight variations in the BGCs in the *C. destructans* isolates could be due to variations in the quality of the assemblies, which is indicated by the different assembly quality metrics (Table 1). Misassembles, particularly from short-read data, can lead to skipping or duplication of NRPS or PKS. Moreover, it can lead to swaps that obscure the true order of genes or protein domains affecting the analysis of BGCs^70^. There were some predicted compounds linked to BGCs unique to *C. fimbriata*, and *C. albifundus* (Table 2). This is probably because these are completely different species from *C. destructans*, with different genomic makeup (Fig. 5), as well as different niches. Based on the MIBiG, all the *Ceratocystis* genomes had an NRPS BGCs which encode the compound dimethylcoprogen (Table 2). Dimethylcoprogen is an extracellular siderophore encoded by NRPS which is a conserved virulence determinant of plant pathogenic ascomycetes^69^. Deletion of NPS6, which encodes NRPS responsible for dimethylcoprogen resulted in reduced virulence and hypersensitivity to hydrogen peroxide of the phytopathogens *Cochlibolus miyabeanus*, *F. graminearium*, and *Alternaria brassicola*^75^.

### Cytochrome P450s, transporters, and putative effectors

The *C. destructans* isolates had between 37 and 46 detected cytochrome P450s in their genomes (Table 3). The cytochrome P450s consisted of the P450 class names: E-class P450, CYP2D; P450, CYP52; and E-class P450, group IV which were identified to be 1, 6, and 1, respectively in all the *C. destructans* isolates (Supplementary Data 1). In addition, the classes cytochrome P450, P450, group I, and pisatin demethylase-like were also identified but with different numbers among the isolates (Supplementary Data 1). Pisatin demethylase, a cytochrome P450, is involved in demethylation of pisatin (phytoalexin) into a non-toxic product^76^. This demethylation of phytoalexin by fungi has been established to be important in pathogenicity, with some exceptions there is a correlation between phytoalexins tolerance and host range^77^. *C. destructans*, *C. fimbriata*, and *C. albifundus* have different hosts, suggesting that their pisatin demethylase might differ. Indeed, cytochrome P450 families (CYP5078A4b and CYP620F1b) were unique to *C. destructans* isolates. In contrast *C. albifundus*, and *C. fimbriata* had CYP5150A2, and CYP5037B3, respectively, as unique families belonging to pisatin demethylase class (Supplementary Data 1). Supplementary Data 1 also provides details on all the cytochromes which includes putative names, and best hit in the Nelson’s database.

Proteome annotated with the NCBI database identified 241 and 247 *transporters* for *C. albifundus* and *C fimbriata*, respectively (Table 3). These were verified by querying the transporter classification database (TCDB) using default settings. In contrast, *C. destructans* isolates had between 248 and 254 transporters, respectively. The identified transporters included proteins which had a pathogenesis GO annotations (Supplementary Data1), of which we already described some of their roles. We also managed to identify 57.4 ± 2.1 putative effector proteins in the *C. destructans* isolates (Table 3). These include some of the pathogenesis features we already describe such as 1-phosphatidylinositol phosphodiesterase, as well as a number of CAZymes. Supplementary Data 1 provides the sequences for proteins predicted to be effectors, including their localization. Total predicted effectors for *C. albifundus* (62) were not significantly higher than those of *C. destructans* (Table 3). In contrast, *C. fimbriata* had almost 40% more predicted effectors (Table 3). The results of our predicted effectors were comparable to that of Fourie et al.^32^. Moreover, we identified 15 putative effectors unique to *C. destructans* (based on phylogenetic analysis; Supplementary Fig. 2). Their annotation and hits in the PHI-database are provided in Supplementary data 1. These effectors might be crucial for the adaptation of *C. destructans* to almonds.

## Conclusion

In this work, we presented the first high quality draft genome sequences of five *C. destructans* isolates isolated from Ceratocystis canker-infested almond trees. We incorporated a number of bioinformatics tools and predicted protein-coding genes of the assembled genomes. From the predicted proteomes, we annotated for CAZymes, cytochrome P450S, transporters, and secondary metabolite gene clusters (BGCs), all of which are important virulence factors for canker pathogens. Moreover, we identified 57.4 ± 2.1 putative effector proteins; effector proteins are known to play critical roles like evasion of the host immune system, facilitating the success of phytopathogens. We also performed Gene Ontology (GO) annotations and provided detailed descriptions of the genes with a pathogenesis annotation. GO leverages domain-specific ontologies to give comprehensive functional information on gene products^78^. The primary gene predictions and annotations from the current study will serve as a valuable resource for *in planta* expression profiling, molecular information required for targeted knock-out mutations, as well as genetic diversity studies. There is still a need to experimentally validate the predicted genes and transcriptomic experiments could add depth to the resource we presented. In addition to validating the existence of predicted genes, transcriptomics can also help improve annotations and the identification of the intro-exon structures of predicted genes. With *C. destructan*s being likely endemic to California, further sequencing of other *C. destructans* isolates could help to estimate the pan-genome and genomic heterogeneity/homogeneity of this species. Such information will be critical to establish structural variations in the population of this pathogen and determine how they might correlate with host adaptations. Though the presented genomes are of good quality (based on BUSCO, genome size, and total predicted proteins), combining long reads and short read sequencing technologies will likely reduce the number of contigs, which is crucial for better annotations. More work still needs to be done; however, the present study provides the foundation for future investigations on this important almond canker pathogen.

## Methods

### Biological material, genomic DNA extraction, and sequencing

*Ceratocystis destructans* isolates KARE979, KARE1622, KARE1624, KARE1428, and KARE490 were isolated from almond trees exhibiting typical Ceratocystis canker symptoms^4,5^. A detailed description of the isolation procedure, orchard location, and year of isolation was provided by Holland et al.^5^. For whole genome sequencing of the isolates, genomic DNA was isolated from hyphal tip purified isolates grown in 250-ml Erlenmeyer flasks containing 100 ml of potato dextrose broth (PDB; Difco Laboratories) and incubated for 7 days at 25 °C and 150 rpm. Approximately 100 mg of mycelia were vacuum filtered from the PDB cultures and collected into 2 ml micro-centrifuge tubes and frozen in liquid nitrogen; mycelia were ground while still frozen. DNA extraction was done with a modification of the Cetyltrimethylammonium Bromide (CTAB) protocol according to Morales-Cruz et al.^12^. The quality and the integrity of the isolated DNA were measured using the NanoDrop spectrophotometry (ND-100, NanoDrop Technologies, Inc, Wilmington, DE, USA), and agarose gel electrophoresis, respectively. In preparation for sequencing, DNA was fragmented by sonication using a Bioruptor (NGS; Diogenade, Liège, Belgium). DNA libraries were prepared using the KAPA library preparation kit (catalog number KK8201, KAPA Biosystem, Woburn, MA, USA), using 1 µg of genomic DNA as input. Insert size selection was carried out using the Life E-gel SizeSelect (Life Technologies, Carlsbad, CA, USA), and the final library quality and quantity were analyzed using a Bioanalyzer 2100 (Agilent Technologies, CA, USA). Sequencing was carried out at the DNA Technologies core of the University of California, Davis, using a HiSeq2500 machine, generating paired end reads of 150 bp.

### Assembly, and gene prediction

Quality trimming (Q > 20) and adapter removal were carried out using Trimmomatic v. 0.36 with the following parameters: ILLUMINACLIP:TruSeq3-PA.fa:2:30:10 LEADING:3 TRAILING:3 SLIDINGWINDOW:10:20 MINLEN:90^79^. Assembly of the filtered reads was performed using SPAdes^80^ with variable k-mer size. K-mer size of 23 produced the best assembly contiguity (N50) and completeness (BUSCO) and was used for all assemblies. Quality assessments of the assembled genomes were performed using Quality Assessment Tool for Assembled Genomes (QUAST)^81^. The Benchmarking Universal Single-Copy Orthologs (BUSCO) software^33^ was used to assess the completeness of the assembled genomes, benchmarking with fungi_odb10. Before gene prediction, repeat masking was performed using RepeatMasker 4.1.1(RepeatMasker Home Page). Genes were predicted using a combination of Augustus^82^, and GeneMark-ES trained on the genome of *Fusarium graminearium*, following the GenSAS eukaryotic annotation pipeline^83^. For consensus gene predictions, EvidenceModeler (EVM)^84^ was used to compile a consensus gene set from the predictions of Augustus and Genemark-ES.

### Gene content comparison, and annotations

For comparison of the predicted proteins across isolates, the Orthovenn2 server for whole-genome comparison and annotation of orthologous clusters^85^ was used. The Orthovenn2 output also includes Gene Ontology (GO) annotations. From the GO annotations, we searched for the key word “pathogenesis” GO term/annotation. The GO (Gene Ontology Resource) is a comprehensive source of functional information on gene products that leverages domain-specific ontologies^76^. Proteins with a pathogenesis GO annotation were queried against the PHI-database^86^. The genome-based distance matrix calculator (http://enve-omics.ce.gatech.edu/g-matrix/index) was used to calculate average nucleotide identities. For comparative purposes, assembled genomes of *Ceratocystis albifundus* (GCA_002742255.2)^29^, and *Ceratocystis fimbriata* (GCA_000389695)^28^, were included in the analysis. The downloaded assembled genomes of *C. albifundus*, and *C. fimbriata* were subjected to similar pipelines with the same parameters as *C. destructans* isolates to standardize the comparisons. For synteny, D-GENIES^87^ was used to visualize the dot plots using Minimap2^88^ with the default settings, the final plots were filtered for strong precision.

Secondary metabolites gene clusters were predicted using the antiSMASH fungal version^22^. Carbohydrate active enzymes (CAZymes) were predicted using the dbCAN2 a meta-server HMMer-based classification system^89^ using default settings. The fungal cytochrome P450 database (FCPD)^23^ was used to annotate cytochrome P450s from the proteome using the Phylum Ascomycota P450 sequences database (BLASTP e-value ≤ 10; ≥ 44% amino acid identity). Proteins were also annotated using BLAST + ^90^ and the NCBI non-redundant fungi protein database. From the annotated proteins, keyword search “transporter” was used to identify proteins annotated as transporters. To identify putative effectors from the proteome, SECRETOOL’s classical secretion pipeline was used to identify the total secretome^20^.The classical secretion pipeline includes processing the data with TargetP, SignalP, PredGPI, and WoLFSPORT. From the secreted protein pool, EffectorP^25^ was used for the identification of putative effector proteins. For the identified effector proteins, all the protein sequences are provided in Supplementary Data 1.

### Supplementary Information

Supplementary Information 1.

Supplementary Information 2.

Supplementary Information 3.

## Data Availability

All sequence data generated for this work can be accessed via GeneBank under BioProject PRJNA981158 with accession numbers SAMN35663300—SAMN35663304. https://www.ncbi.nlm.nih.gov/biosample/35663300. https://www.ncbi.nlm.nih.gov/biosample/35663301. https://www.ncbi.nlm.nih.gov/biosample/35663302. https://www.ncbi.nlm.nih.gov/biosample/35663303. https://www.ncbi.nlm.nih.gov/biosample/35663304. Biological material (isolates), assemblies and predicted protein files can be provided via requests to the corresponding author.

## Abbreviations

BGC: Biosynthetic gene cluster
CAZymes: Carbohydrate-active enzymes
DNA: Deoxyribonucleic acid
GC-content: Guanine-cytosine content
GO: Gene Ontology
Kbp: Kilo base pairs
Mbp: Mega base pairs
MIBiG: Minimum Information about a Biosynthetic Gene cluster
