# Supplementary material for: Whole genome sequencing and analysis of multiple isolates of Ceratocystis destructans, the causal agent of Ceratocystis canker of almond in California
Source: Sci Rep. 2023 Sep 8;13:14873. doi: 10.1038/s41598-023-41746-6 (PMC10491840; doi:10.1038/s41598-023-41746-6)
Supplement: Supplementary file 3 — Supplementary Information 3. [file 41598_2023_41746_MOESM3_ESM.pptx]

## Slide 1
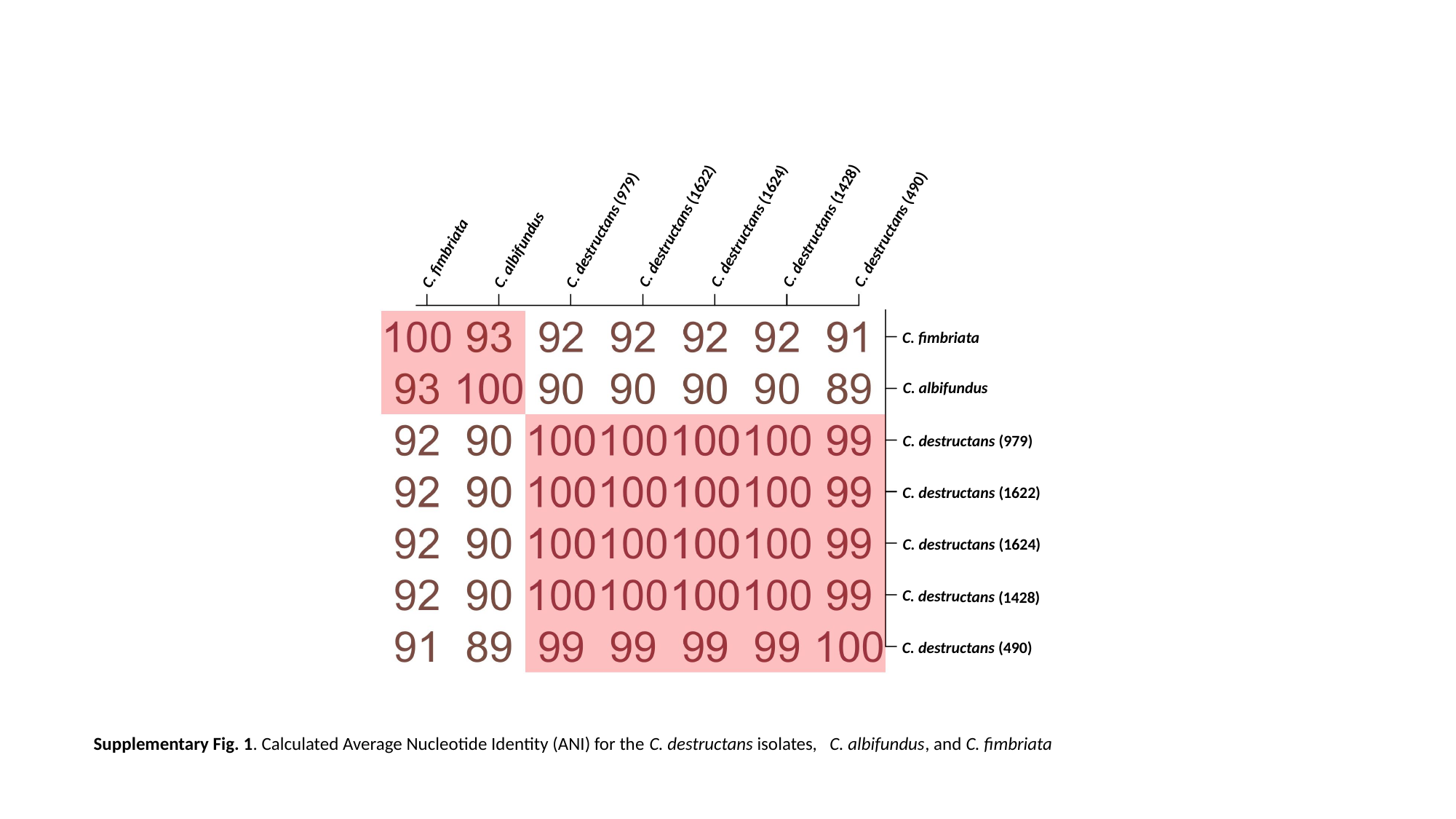

C. destructans (1428)
C. destructans (490)
C. destructans (1624)
C. destructans (1622)
C. destructans (979)
C. albifundus
C. fimbriata
C. fimbriata
C. albifundus
C. destructans (979)
C. destructans (1622)
C. destructans (1624)
C. destructans (1428)
C. destructans (490)
Supplementary Fig. 1. Calculated Average Nucleotide Identity (ANI) for the C. destructans isolates, C. albifundus, and C. fimbriata

## Slide 2
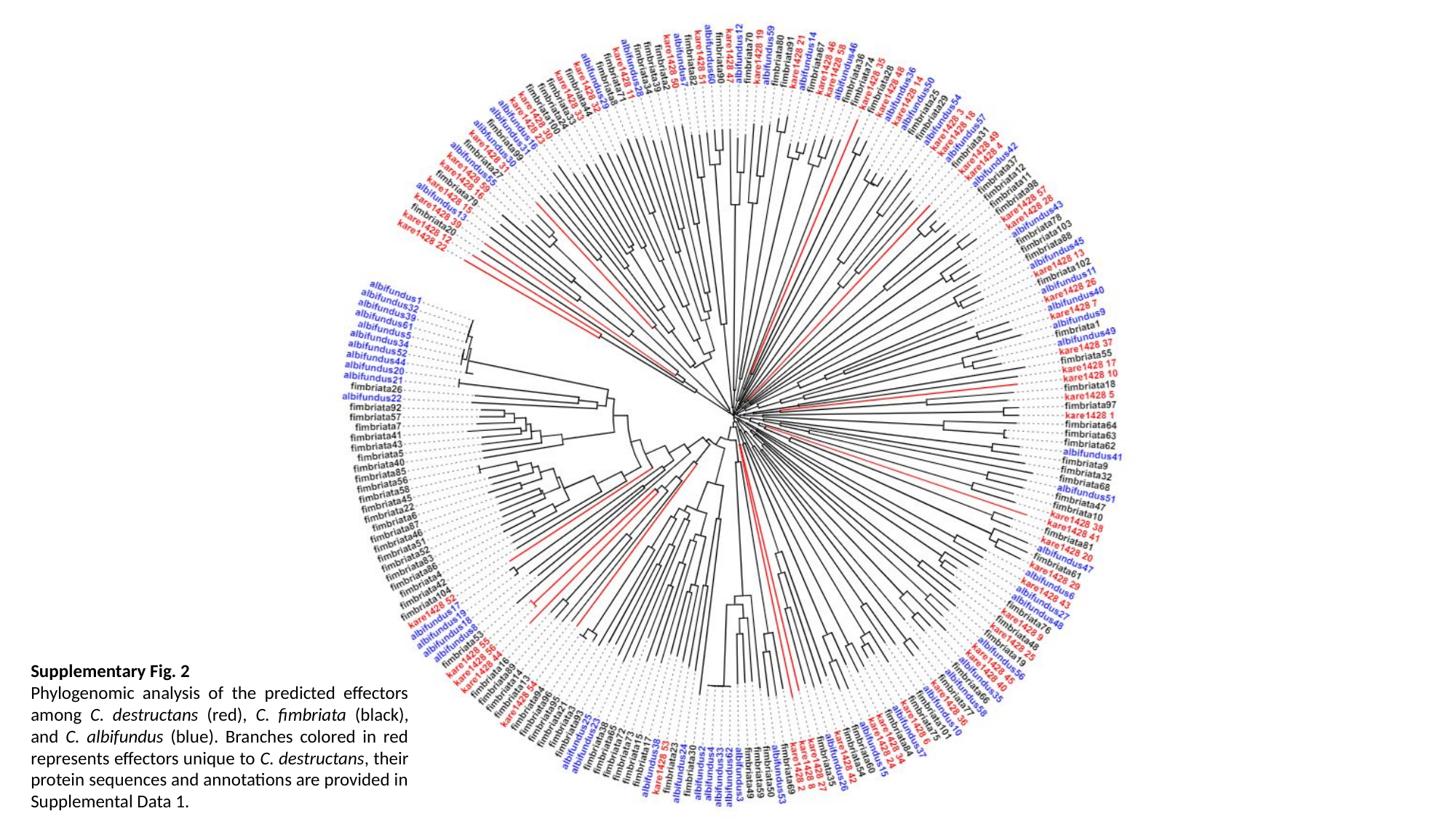

Supplementary Fig. 2
Phylogenomic analysis of the predicted effectors among C. destructans (red), C. fimbriata (black), and C. albifundus (blue). Branches colored in red represents effectors unique to C. destructans, their protein sequences and annotations are provided in Supplemental Data 1.

## Slide 3
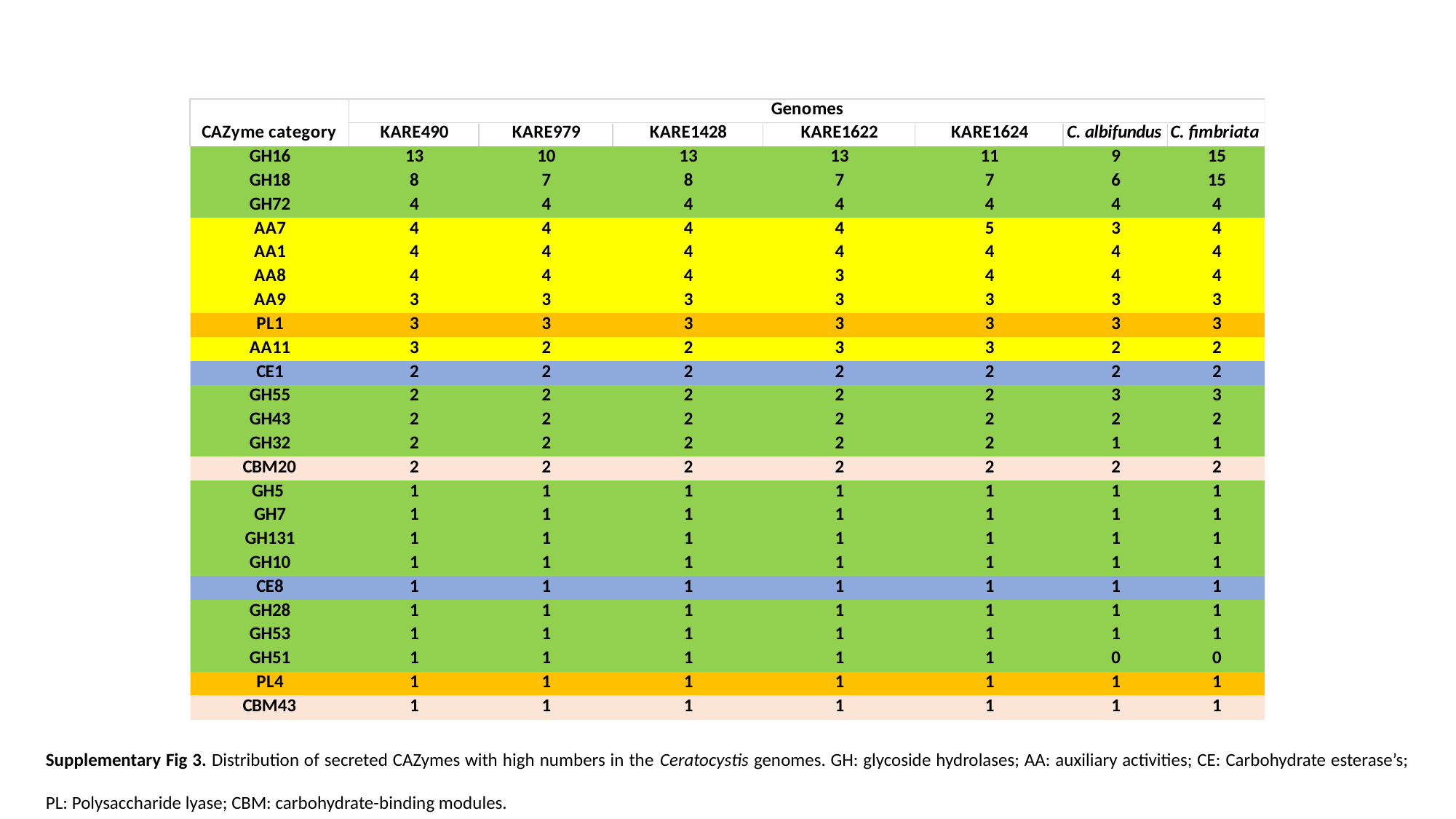

Supplementary Fig 3. Distribution of secreted CAZymes with high numbers in the Ceratocystis genomes. GH: glycoside hydrolases; AA: auxiliary activities; CE: Carbohydrate esterase’s; PL: Polysaccharide lyase; CBM: carbohydrate-binding modules.
